# Improving the global understanding of CADASIL‐ a monogenic cause of vascular dementia

**DOI:** 10.1002/alz.088571

**Published:** 2025-01-03

**Authors:** Danit G Saks, Perminder S. Sachdev, AusCADASIL Study Group, the Global CADASIL Consortium (GCC)

**Affiliations:** ^1^ Centre for Healthy Brain Ageing (CHeBA), University of New South Wales, Sydney, NSW Australia; ^2^ Neuropsychiatric Institute, Prince of Wales Hospital, Randwick, NSW Australia; ^3^ Centre for Healthy Brain Ageing (CHeBA), University of New South Wales, UNSW Sydney, NSW Australia

## Abstract

**Background:**

Cerebral autosomal dominant arteriopathy with subcortical infarcts and leukoencephalopathy (CADASIL) is a rare, hereditary cerebrovascular disease which causes stroke, complex migraine, and cognitive impairment. Given its monogenic nature, CADASIL is considered a ‘pure’ model of small vessel disease and vascular dementia. CADASIL is caused by *NOTCH3* pathogenic variants with a broad resulting phenotypic spectrum. There are few CADASIL cohort studies worldwide, often with small or moderate samples, such that any study protocol variation limits the comparability of conclusions across populations. Our recently established Global CADASIL Consortium (GCC) will collate current research and develop guidelines for harmonised protocols and minimum datasets. Further, we have begun the first Australian cohort study of CADASIL, AusCADASIL, to determine the phenotypic presentation, progression, and pathogenic variants in this population.

**Method:**

Literature search, publicising at the 2023 VASCOG conference, and chain‐referrals were used to identify CADASIL researchers and experts who may be interested in contributing to the global collaborative effort. Study meta‐data sharing is ongoing including demographics, clinical, genetic, and imaging outcomes.

AusCADASIL will include 150 participants with genetically‐confirmed CADASIL, family history of CADASIL or suspected CADASIL symptoms, and 150 *NOTCH3*‐negative controls; to be assessed at six main sites. Participants will complete a comprehensive medical, neuropsychological, neurological, ocular, blood biomarker and genetic assessment, including annual follow‐up for 4 years to monitor progression. Data from this cohort will contribute to the international consortium.

**Result:**

The GCC, comprised of 75 individuals from 18 countries, has begun by sharing meta‐data from 10 cohorts based in Australia, East Asia, Netherlands, USA, Spain, France, United Kingdom, and Finland with recruited sample sizes ranging from 20‐520 individuals, and discussing development of working groups. Recruitment for AusCADASIL opened in November 2023 and assessments will begin in early 2024.

**Conclusion:**

There is increased global interest in CADASIL as a model for small vessel disease and vascular dementia. This recently established international body is committed to improving the understanding of CADASIL, harmonising assessment protocols and pooling data for larger analyses of biomarkers. AusCADASIL is the first Australian cohort study of CADASIL and will contribute to international efforts.

